# Carbon Nanomaterials for Multifunctional Applications

**DOI:** 10.3390/ma19102064

**Published:** 2026-05-15

**Authors:** Ying Wu

**Affiliations:** School of Materials Science and Engineering, University of Science and Technology Beijing, Beijing 100083, China; wuying@ustb.edu.cn

## 1. Introduction

Carbon-based nanomaterials have evolved from the study of low-dimensional systems (e.g., graphene [1], carbon nanotubes [2], and fullerene [3]) into diverse functional materials engineered for practical applications. In recent years, research has shifted from exploring intrinsic properties to tailoring structure–property relationships through controlled fabrication, structural design, and interfacial engineering [4,5,6]. This transition is particularly evident in applications such as flexible electronics, electromagnetic shielding, energy storage, thermal management, and advanced manufacturing, where performance is governed not only by the intrinsic properties of carbon nanostructures but also by their organization across multiple length scales [7,8,9,10,11,12]. To translate nanoscale properties into device-level functionality, significant efforts have been devoted to developing diverse material systems (such as functionalized derivatives [13,14,15], hybrids [16,17], and composites [18]) and to constructing various structural forms (such as films [19], fibers [20], foams [21], aerogels [22], patterns [23], and fabrics [24]). Such architectures enable the integration of structural and functional features across different scales, thereby enhancing the overall performance of carbon-based materials in practical applications.

Despite these progresses, several key issues continue to limit the practical implementation of carbon-based nanomaterials. Importantly, these challenges are no longer isolated problems in synthesis or characterization, but are increasingly interconnected and application-driven. In the context of the contributions collected in this Special Issue, three aspects are particularly critical.

First, controllable fabrication and structural uniformity remain challenging, especially for the large-scale fabrication of large-area or bulk materials [15,25,26]. Although techniques such as chemical vapor deposition, laser-induced conversion, and solution-based assembly have been widely developed, achieving consistent microstructure (e.g., defect density, layer continuity, and porosity) across different length scales is still difficult. This directly affects the reproducibility and scalability of carbon-based systems.

Second, rational structural design for multifunctional performance is still largely empirical. Many high-performance systems rely on hierarchical or heterogeneous architectures (e.g., layered structures, porous networks, or hybrid composites), where properties emerge from the coupling of multiple structural features [27,28,29]. However, quantitative relationships between structural parameters and macroscopic performance are not yet well established, making it difficult to generalize design strategies across different applications.

Third, interfacial and structure–property characterization under realistic conditions remains insufficient [30,31]. Advanced techniques have provided valuable insights into microstructure and local interactions, but linking these observations to device-level performance, especially under dynamic or service conditions, remains a challenge. In particular, interfacial effects such as charge transfer, stress transfer, and structural evolution are often inferred rather than directly quantified.

This Special Issue addresses these challenges by bringing together recent studies that emphasize structure–property relationships through controlled fabrication, structural design, and advanced characterization of carbon-based systems. The accepted contributions cover a range of topics, including large-area graphene characterization, hierarchical conductive networks, layered architectures for electromagnetic shielding, laser-induced graphene fabrication, and interface-related mechanisms in composites. These works highlight current strategies for improving structural control, understanding functional mechanisms, and advancing the practical application of carbon nanomaterials.

## 2. An Overview of Published Articles

Manuscripts revealing the structure–property relationships of carbon-based materials through advanced characterization and design strategies were submitted to this Special Issue. After peer reviews, seven papers were finally accepted for publication.

Contribution 1 [32] investigated the non-destructive characterization of large-area CVD monolayer graphene on silicon using terahertz emission spectroscopy and microscopy. The dynamic electronic properties were studied by comparing terahertz signals from graphene on different silicon substrates, with and without oxide layers. The results revealed that terahertz emission is highly sensitive to variations in the graphene–silicon heterojunction, enabling clear distinction between substrate conditions. The authors established a mechanism based on interfacial charge transfer and built-in electric fields, and obtained large-area spatial mapping of graphene quality. The work demonstrated a rapid, non-contact, and high-sensitivity approach for evaluating graphene interfaces, with strong potential for semiconductor-compatible device characterization.

Contribution 2 [33] investigated the synthesis of nitrate-doped polypyrrole/silver nanowire and the effect of controllable polypyrrole shell thickness on structure and performance. The authors explored the formation of a three-dimensional porous conductive network assembled from one-dimensional core–shell nanostructures and revealed that such an architecture effectively reduces internal resistance while maintaining structural stability. The results established that the synergistic effect of Ag nanowires and polypyrrole improves electrochemical performance, and high capacitance with good stability was obtained, demonstrating the effectiveness of the designed structure.

Contribution 3 [34] developed a novel two-layer graphene nonwoven fabric for electromagnetic interference shielding applications. The authors studied the structural design consisting of a porous fibrous upper layer and a dense conductive lower layer, and explored the synergistic effects of this layered architecture on shielding performance. The results revealed that the combination of reflection, multiple internal reflections, and absorption significantly enhances EMI shielding effectiveness. This study demonstrated that rational structural design of graphene-based fabrics is an effective strategy for developing high-performance EMI shielding materials.

Contribution 4 [35] reported the fabrication of SiC/(Hf_0.25_Ta_0.25_Zr_0.25_Nb_0.25_)C/C high-entropy ceramic nanocomposites using a polymer-derived ceramic method combined with spark plasma sintering. The authors studied the polymer-to-ceramic transformation process and explored the microstructural evolution and phase formation at different temperatures. The results revealed that the phase composition and microstructure can be tuned by precursor design and heat treatment, leading to the formation of high-entropy carbide phases at elevated temperatures. This study established that introducing transition metal components enhances ceramic yield and structural stability, and improved mechanical properties were obtained, demonstrating the effectiveness of the designed nanocomposite system.

Contribution 5 [36] explored the inter-ply slipping behavior of carbon fiber-reinforced epoxy prepregs during hot diaphragm preforming using a dedicated experimental system. The authors studied the effects of temperature and viscosity, and explored their relationship with wrinkle formation. The results revealed a three-stage slipping mechanism and showed that higher temperatures reduce slipping resistance. This study established a predictive model for inter-ply slipping and wrinkle formation, and improved simulation accuracy was obtained, demonstrating its effectiveness for optimizing forming processes.

Contribution 6 [37] investigated laser-induced graphene (LIG) formation from polymers using CO_2_ and UV lasers. The authors studied the influence of laser parameters and explored their effects on structure and morphology. The results revealed that laser type and processing conditions strongly influence the microstructure, porosity, and crystallinity of LIG, with CO_2_ and UV lasers producing distinct morphologies. The study established that optimized laser parameters improve graphene quality and surface properties, and well-defined patterned graphene structures were obtained. This work demonstrated an effective and controllable strategy for fabricating LIG with tunable properties for potential applications in sensors and functional devices.

Contribution 7 [38] investigated the purification and preparation of graphene-like nanoplates from natural microcrystalline graphite using flotation, hydrothermal treatment, and acid leaching. The authors studied the effects of grinding time and reagent dosage on graphite recovery and purity, and explored the structural transformation during chemical treatment. The results revealed that optimized flotation conditions significantly improve graphite concentration, while subsequent chemical treatments disrupt layer stacking and promote the formation of graphene-like nanoplates. This study established an effective route for producing high-purity graphene-like nanoplates, demonstrating their potential for advanced applications.

## 3. Summary

This Special Issue, ‘Carbon Nanomaterials for Multifunctional Applications’, collects recent research contributions focusing on the design, fabrication, characterization, and application of carbon-based nanomaterials, including graphene, graphene-derived structures, and other carbon materials. The published works cover a wide range of topics, such as interfacial engineering, hierarchical structural design, and performance optimization for applications in energy storage and electromagnetic shielding, as well as advanced manufacturing. These studies promote the understanding of structure–property relationships and establish effective strategies for enhancing material performance.
